# Correction: Holte et al. A Semi-Automatic Approach for Holistic 3D Assessment of Temporomandibular Joint Changes. *J. Pers. Med.* 2023, *13*, 343

**DOI:** 10.3390/jpm14121159

**Published:** 2024-12-19

**Authors:** JPM Editorial Office

**Affiliations:** MDPI AG, Grosspeteranlage 5, 4052 Basel, Switzerland; jpm@mdpi.com

The journal’s Editorial Office and Editorial Board are jointly issuing a resolution and removal of the Journal Notice linked to this article [1], as well as an update to the original publication. Following concerns raised about the integrity of the peer-review, the Editorial Office has conducted a post-publication peer-review of this article. This process included the recruitment of a new independent reviewers and was supervised by the Editor-in-Chief of the journal to ensure full compliance with MDPI’s Editorial Process (https://www.mdpi.com/editorial_process).

As a result of this process, Editor-in-Chief and the authors have agreed to update the following aspect of this publication:

Reviewer report 1 was removed from the peer review record and a new report was uploaded (https://www.mdpi.com/2075-4426/13/2/343/review_report).

With this update, the Editor-in-Chief is satisfied that the Editorial Process relating to this article has been completed as per MDPI’s Editorial Process policy. The Editorial Office would like to thank the authors for their collaboration during this process.
