# Supplementary figures and images for: A Semi-Automatic Approach for Holistic 3D Assessment of Temporomandibular Joint Changes
Source: J Pers Med. 2023 Feb 16;13(2):343. doi: 10.3390/jpm13020343 (PMC9959062; doi:10.3390/jpm13020343)

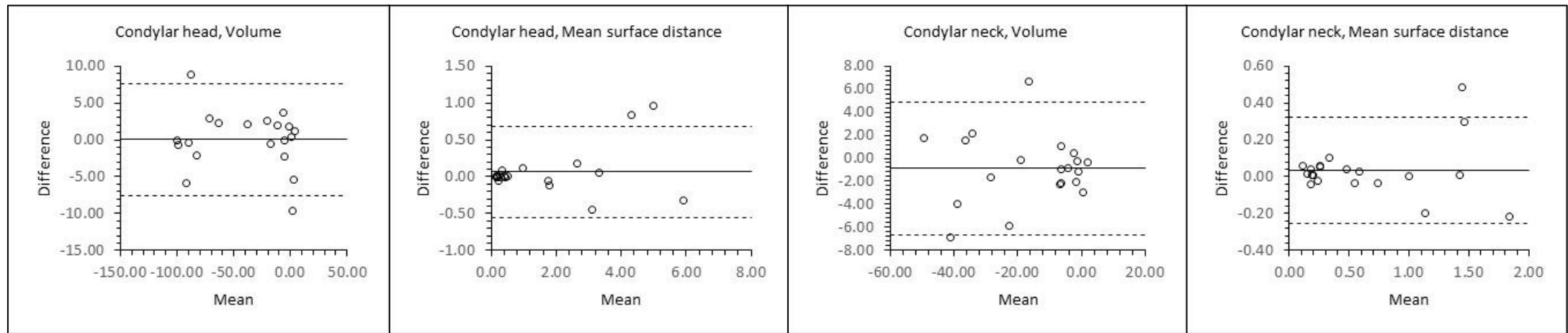

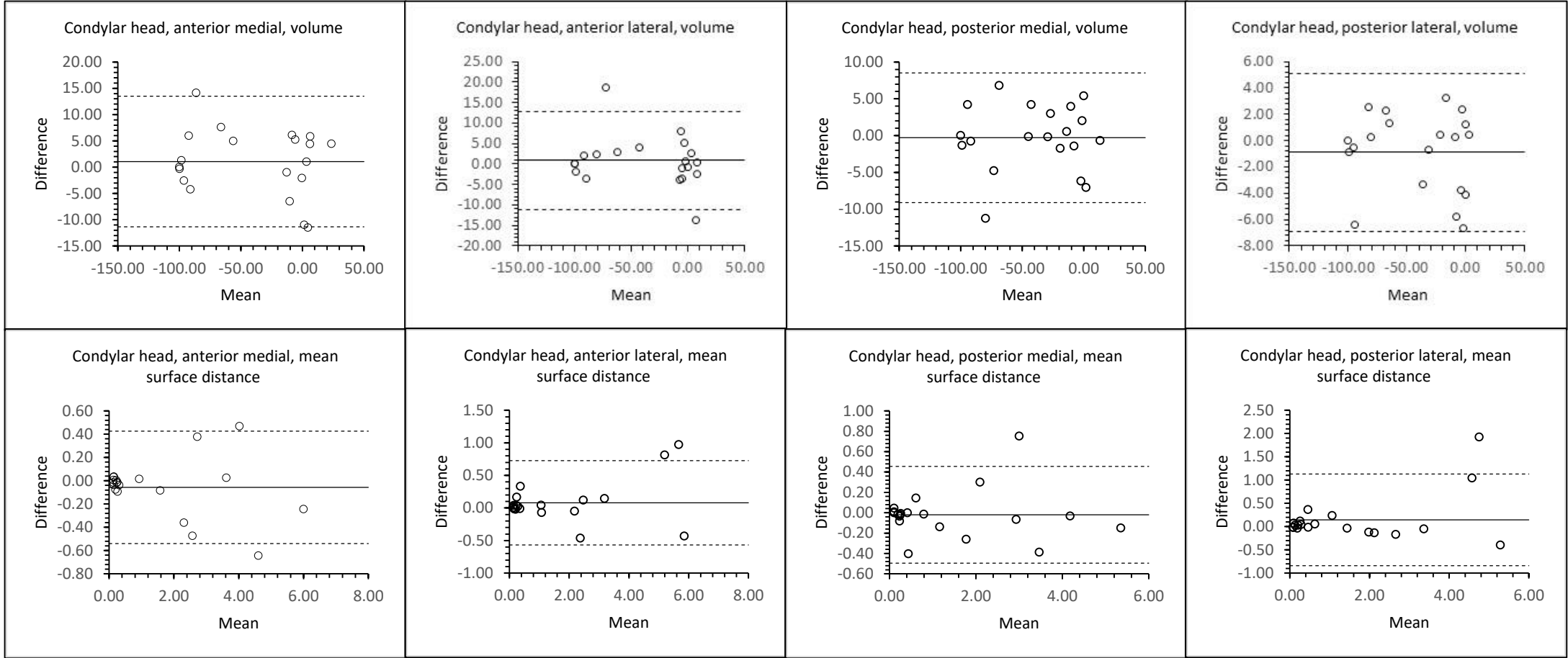

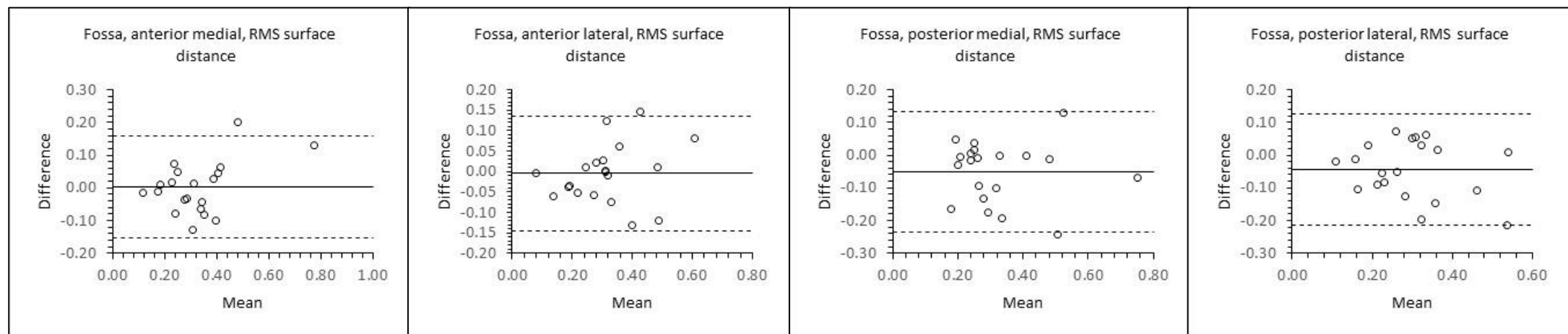

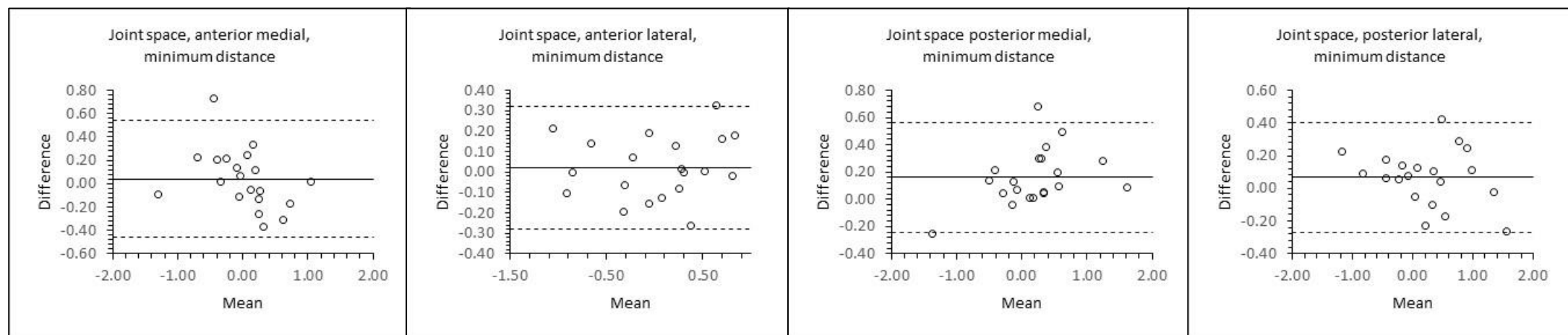

Supplement: Supplementary file 1 [file jpm-13-00343-s001.zip › jpm-2182577-supplementary.pdf]
